# A retrospective study of 21 cases of malignant odontogenic tumours from two tertiary health centres in Nigeria

**DOI:** 10.11604/pamj.2015.20.371.6611

**Published:** 2015-04-15

**Authors:** Ahmed Oluwatoyin Lawal, Olujide Oladele Soyele, Akindayo Olufunto Akinyamoju

**Affiliations:** 1 Department of Oral Pathology, College of Medicine, University of Ibadan, Ibadan, Nigeria; 2 Department of Oral Maxillo-facial Surgery and Oral Pathology, Obafemi Awolowo University, Ile-Ife, Nigeria

**Keywords:** Malignant odontogenic tumours, ameloblastic carcinoma, ameloblastic fibrosarcoma.

## Abstract

**Introduction:**

Malignant odontogenic tumours (MOTs) are relatively rare tmours and only few cases have been reported in the sub-Sahara African literature. The aim of this study was to describe the demographic distribution of malignant odontogenic tumours in two tertiary health centres based on the current WHO 2005 classification scheme.

**Methods:**

We reviewed 21 malignant odontogenic tumours out of a total of 374 odontogenic tumours from two Tertiary Health Centres. Information regarding histology, location, patients age and gender for MOTs were analysed using SPSS for Windows (version 20.0; SPSS Inc. Chicago, IL).

**Results:**

Twenty one (5.6%) MOTs out of a total of 374 odontogenic tumours were seen from the two institutions over the study period. The median age for MOTs was 42.0 (±19.0) years (range = 16-66 years). The male: female ratio was 2.5:1 and 85.7% occurred in the mandible. Ameloblastic carcinoma (AC) with 13 (61.9%) cases was the most common MOT. AC had a mean age of 37.5 (±11.9) years. AC had a mandible: maxilla ratio of 5.5:1 with majority (84.6%) occurring in the posterior mandible.

**Conclusion:**

This study showed that MOTs are rare lesions. AC was the most common MOT and majority of MOTs occurred in the posterior mandible of male patients. The study helps to better elucidate the demography of MOTs in sub-Sahara Africans.

## Introduction

Malignant odontogenic tumours (MOTs) are rare primary head and neck lesions that arise within the mandible and maxilla [1]. Although, the relative frequency of odontogenic tumours (OT) vary from continent to continent [2, 3], most authors agree that MOTs are rare tumours accounting for between 0 and 6.1% of all OTs [4]. Similar to benign OTs, MOTs develop from either epithelial or mesenchymal components with the carcinomas exhibiting an overwhelming predominance over the sarcomas [4, 5]. According to recent WHO 2005 classification of OTs, the carcinomas include metastasizing (malignant) ameloblastoma (MA), ameloblastic carcinoma (AC), primary intraosseous squamous cell carcinoma (PIOSCC), clear cell odontogenic carcinoma (COCC) and ghost cell odontogenic carcinoma (GCOC). On the other hand, the odontogenic sarcomas include ameloblastic fibrosarcoma, fibro-dentinosarcoma and fibro-odontosarcoma [1, 6]. The rarity of MOTs, variations in their pathogenesis, clinic-pathological features and biological behaviour all contribute to the difficulty in diagnosing these lesions. Furthermore, some MOTs may be difficult to differentiate from their benign counterparts and occasional difficulty in confirming their odontogenic origin may also contribute to the challenge of diagnosing MOTs [7]. Reports on MOTs are sparse especially in sub-Saharan Africa and many are either case reports or reports on one type of MOT. This study, therefore, aims to describe the demographic distribution of MOTs in two tertiary health centres in Nigeria.

## Methods

This study reviewed odontogenic tumours from the Oral Pathology Department, University College Hospital (UCH) over a 21 year period (1990-2011) and the Department of Oral Maxillofacial Surgery/Oral Pathology, Obafemi Awolowo University Teaching Hospital (OAUTH) over a 10 year period (2003-2013). Odontogenic tumours designated as MOTs according to WHO (2005) classification of OTs were selected. Information regarding histology, location, patients age and gender for MOTs were analysed using SPSS for Windows (version 20.0; SPSS Inc. Chicago, IL).

## Results

A total of 374 OTs were diagnosed from the two institutions over the study periods, out of which 21 (5.6%) were diagnosed as MOTs. Table 1 summarises the demography of MOTs in this study. The median age of occurrence for MOTs was 42.0 (±19.0) years (range = 16-66 years) while the peak age incidence was in the fourth decade of life. There was an obvious male preponderance with a male: female ratio of 2.5:1 and majority of cases (85.7%) occurred in the mandible with a mandible: maxilla ratio of 6:1. Ameloblastic carcinoma (AC) with 13 (61.9%) cases was the most common MOT, followed by Primary interosseous squamous cell carcinoma (PIOSCC) with 7 (33.3%) cases while one case (4.8%) of ameloblastic fibrosarcoma (AFS) was seen. Table 2 shows the age distribution of MOTs. AC with a mean age of 37.5 (±11.9) years had a peak age incidence in the 4th decade of life and 69.3% of all cases were seen in the 4th and 5th decades of life. AC occurred predominantly in the mandible with mandible: maxilla ratio of 5.5:1 with majority (84.6%) occurring in the posterior mandibular area. AC also had an obvious male preponderance with a male: female ratio of 3.3:1. PIOSCC occurred at a mean age of 63.6 (±18.6) years (range = 34-82 years) with peak age incidence in the eight decade of life. PIOSCC was seen exclusively in the mandible with 71.4% occurring in the posterior mandible and had an obvious male preponderance with male to female ratio of 2.5:1. There was a statistically significant difference in the mean ages of AC and PIOSCC (t= -3.84, p = 0.001).

**Table 1:** Demographic distribution of 21 cases MOTs

MOT	Sex N (%)	Site	Mean Age	Age range	Peak Age
All MOTs	M, 15 (71.4); F, 6 (28.6)	AMan, 2 (9.5); PMan,16(76.2); Amax, 1(4.8); PMax, 2 (9.5)	42.0	16-82	30-39
AC	M, 10 (76.9); F, 3 (23.1)	PMan, 11(84.6); Amax, 1 (7.7); Pmax, 1 (7.7)	37.5	16-60	30-39
PISCC	M, 5 (71.4); F, 2 (28.6)	AMan, 2 (28.6); PMan, 5(71.4)	63.6	34-82	70-79
AFS	F, 1 (100)	P Max	28.0	-	-

MOT= Malignant odontogenic tumour, AC = ameloblastic carcinoma, PISCC= Primary Intraosseous squamous cell carcinoma, Ameloblastic fibrosarcoma, M = male, F = female, AMan = Anteriror mandible, PMan= Posterior mandible, Amax = Anterior maxilla, PMax= Poterior Maxilla

**Table 2:** Age group distribution of MOTs

Age group	N (%)
0-9	0(0)
10-19	1(4.8)
20-29	2(9.5)
30-39	7(33.3)
40-49	3(14.3)
50-59	3(14.3)
60-69	1(4.8)
70-79	3(14.3)
80-89	1(4.8)
Total	21(100)

## Discussion

This study showed that out of a total of 374 OTs from the two centres, 21 MOTs representing 5.6% of OTs were seen. MOTs have been reported to be rare tumours and represent 0-6% of all OTs [8]. It has been suggested that there may be ethnic /geographical variations in the occurrence of MOTs (Table 3) with reports from the Americas mostly reporting that MOTs represent less than 1% of all OTs [2, 9] while studies from Asia and Africa reported that between 2.7%-6.1% of OTs were diagnosed as MOTs [10, 11]. The fact that ACs may arise from dedifferentiation of long standing ameloblastoma which are more likely to be seen in Africans and Asians, may partly explain the higher relative frequencies of MOTs in these regions. It is also possible that genetic and or environmental factors may play a role in the higher occurrence of MOTs in Africans and Asians but this is yet to be substantyiated. The median age of occurrence for MOTs was 42.0 years with an age range of 16-66 years. This was similar to the report of Martinez and Mosqueda who reported mean ages of 41.44 years and 43.9 years respectively [4, 7]. Previous reports have shown that MOTs are predominantly seen in males [8] and this was in conformity with our finding that showed an obvious male predilection. On the contrary, Martinez et al [7] reported no gender predilection in their study of 25 MOTs in Brazilians. MOTs appear to present predominantly in the mandible and the mandibular preponderance obtained from this study (85.7%) was in conformity with reports from previous studies with authors reporting that between 65%-84% of their cases presented in the mandible [7, 8, 12]. Some authors have suggested that the persistence of the prefunctional dental lamina, with potential to develop into dental tissues and tumours in the mandible, especially in the posterior mandible, may in part explain the marked preference of MOTs for the mandible [13]. Previous studies have shown that carcinomas were more common MOTs compared to the sarcomas with some authors reporting that carcinoma accounted for up to 95% of MOTs [10]. This was in consonance with our finding which showed that 95.4% of cases were odontogenic carcinomas with only one case of odontogenic sarcoma seen. However, Martinez et al [7] in a study in Brazil reported a much lower proportion of odontogenic carcinomas with 76% of the 25 reported cases being odontogenic carcinomas while Mosqueda et al [4] reported that 85.4% of the 7 cases from Mexico were odontogenic carcinomas.

**Table 3:** Comparing demography of MOTs from various studies

Country/Year	No of Cases	% of OTs	Carcinomas (%)/Sarcomas (%)	Age range/Mean Age (years)	M:F	Man: Max	Commonest MOT (%)
Mexico/2003^4^	7	2.2	6(85.7)/1(14.3)	25-72/43.8	5:2	6:1	MA (28.6)
Brazil + Mexico/2014^7^	25	1.2	19 (76)/6 (24)	7-77/41.4	12:13	21:4	AC (32.0)
USA/Thailand/2012^1^	17	NA	17 (100)/0 (0)	20-86/50.29	12:5	11:6	AC (29.4)
USA/2004^8^	9	NA	9 (100)/0 (0)	27-81/51.2	7:2	6:3	MA (44.4)
Nigeria/2015^A^	21	5.6	20 (95.2)/1 (4.8)	16-66/45.7	15:6	18:3	AC (61.9)

OTs = Odontogenic tumour, MOT = Malignant odontogenic tumour, AC = Ameloblastic carcinoma, MA= Malignant ameloblastoma, M = Male, F = Female, Man= mandible, Max= maxilla A= present study.

Ameloblastic carcinoma was recently described as a rare primary odontogenic malignancy that combines the histological features of ameloblastoma with cytological atypia. AC exhibit cytologic and/or histologic evidence of malignancy regardless of whether they have metastasized or not [6, 14]. AC was the most common MOT representing 61.9% of MOTs and 65% odontogenic carcinomas seen in this series. This was similar to the findings Martinez et al [7] who reported that 19 of 25 (76%) cases of MOT were carcinomas and 8 of the 19 (42.1%) carcinomas were ACs. AC has been reported to be predominant in the mandible as supported by this study which showed an 84.6% mandibular presentation. However, Yoon et al [15] reported a 60% maxillary presentation from China, although the small sample of 6 cases they presented might have affected their results. The World Health Organization (WHO) 2005 defined Primary intraosseous squamous cell carcinoma (PIOSCC) as a central jaw carcinoma derived from odontogenic epithelial remnants [6]. PIOSCC was first described by Loos in 1913 [16] as central epidermoid carcinoma of the jaw and diagnostic criteria for PIOSCC include; the absence of initial connection with the overlying mucosa or skin and exclusion of metastasis from a distant primary tumour by physical and radiographic examination during at least a 6-months follow-up period [17, 18]. The mean age of occurrence for PIOSCC in this study was 63.6 years which was higher than then 56.2 years and 54 years obtained by Chaisuparat et al [16] from USA and Huang et al [19] from China respectively. The reason for the higher mean age in our series is not immediately apparent, though Bodner et al [20] in a review of 116 reported cases got a mean age of 60.2 years. It is possible that the criteria used for diagnosing PIOSCC varied in these different studies. The finding of a mandibular predilection was similar to other studies [16, 19, 21] and Martinez's finding of an exclusive mandibular presentation [7] was corroborated by this present study. Only one case of Ameloblastic fibrosarcoma, presenting in the maxilla of a 28 year old female was seen in this series. Previous studies have reported varying demography for AFS, probably because most were small series or case reports. Martinez et al [7] reported a mean age of 19 years while others reported a mean age of 27.3 years and AFS is reported to be the most common MOT in paediatric age group [7, 22].

## Conclusion

This study reviewed 21 cases of MOTs and showed that MOTs are rare lesions. AC was the commonest MOT and majority of MOTs occurred in the posterior mandible of male patients. The study has helped to elucidate the demography of MOTs which is quite rare in African literature. Although this study showed a relatively large number of MOTs, only three out the eight recognised MOTs by WHO were seen in this series, therefore limiting the conclusions that could be deduced from this study.
